# Loss of mRNA surveillance pathways results in widespread protein aggregation

**DOI:** 10.1038/s41598-018-22183-2

**Published:** 2018-03-01

**Authors:** Nur Hidayah Jamar, Paraskevi Kritsiligkou, Chris M. Grant

**Affiliations:** 1 Division of Molecular and Cellular Function, School of Biological Sciences, Faculty of Biology Medicine and Health, Manchester Academic Health Science Centre, The University of Manchester, Manchester, M13 9PT UK; 2 School of Biosciences and Biotechnology, Faculty of Science and Technology, Universiti Kebangsaan Malaysia, 43600 Bangi, Malaysia

**Keywords:** Chaperones, Protein aggregation, Translation

## Abstract

Eukaryotic cells contain translation-associated mRNA surveillance pathways which prevent the production of potentially toxic proteins from aberrant mRNA translation events. We found that loss of mRNA surveillance pathways in mutants deficient in nonsense-mediated decay (NMD), no-go decay (NGD) and nonstop decay (NSD) results in increased protein aggregation. We have isolated and identified the proteins that aggregate and our bioinformatic analyses indicates that increased aggregation of aggregation-prone proteins is a general occurrence in mRNA surveillance mutants, rather than being attributable to specific pathways. The proteins that aggregate in mRNA surveillance mutants tend to be more highly expressed, more abundant and more stable proteins compared with the wider proteome. There is also a strong correlation with the proteins that aggregate in response to nascent protein misfolding and an enrichment for proteins that are substrates of ribosome-associated Hsp70 chaperones, consistent with susceptibility for aggregation primarily occurring during translation/folding. We also identified a significant overlap between the aggregated proteins in mRNA surveillance mutants and ageing yeast cells suggesting that translation-dependent protein aggregation may be a feature of the loss of proteostasis that occurs in aged cell populations.

## Introduction

Protein aggregation is the abnormal association of misfolded proteins into aggregated, insoluble protein structures^1^. It is often classified into two general categories: amyloid and amorphous. The amyloid state is a highly structured, insoluble, fibrillar deposit, usually consisting of many repeats of the same protein^2^. It is central to the pathology of many neurodegenerative diseases including Alzheimer’s, Parkinson’s and Huntington’s. Amorphous protein aggregation is the unordered aggregation of proteins, where proteins aggregate without forming a specific higher order structure. The aggregation of amyloid proteins has been more extensively studied since it is implicated in the pathogenesis of several neurological diseases, whereas, less is known regarding amorphous aggregation. It is not clearly established whether aggregation of amorphous proteins is mechanistically and functionally any different to amyloid proteins in terms of impact to the physiological state of the cell. Additionally, whether the proteins in disease settings are in fact amyloid or amorphous has not been well characterised. Proteins may also form amyloid progressively from initial disordered aggregates^3^.

Protein aggregation occurs following protein misfolding, which arises as a consequence of translational errors during stress and ageing conditions, as well as a consequence of mutations or lack of oligomeric assembly partners^4,5^. Such conditions disrupt the native folding of proteins, exposing hydrophobic regions leading to the formation of high molecular weight aggregates. Newly synthesized proteins are particularly vulnerable to misfolding and aggregation which is potentially toxic to cells^6–8^. A wide-range of proteins are prone to aggregation during normal unstressed conditions, with higher protein abundances and higher translation rates suggested as being the main determinants^9–11^. Aggregated proteins are also often substrates of ribosome-associated Hsp70 chaperones indicating that they are likely to be susceptible to aggregation during translation and folding^9^. Cellular stresses appear to reduce the general threshold for protein aggregation resulting in the aggregation of proteins which do not possess distinct kinds of physicochemical properties^9,10,12,13^. Proteins in stress-induced aggregates tend to have lower abundances, slower translation rates and increased sizes relative to unstressed protein aggregates. This suggests that protein aggregation is a normal physiological event, but conditions which act to perturb cellular homeostasis, can increase the burden of protein aggregation^10^. Several aggregation-prone yeast proteins have human homologues that are implicated in misfolding diseases, highlighting that similar mechanisms may apply in disease and non-disease settings. However, the relevance of widespread protein aggregation in disease is unknown.

The process of mRNA decay is recognised as a major contributor to the regulation of gene expression, as well as maintaining regulatory responses crucial for cellular homeostasis. mRNA turnover plays critical roles in assessing the accuracy of mRNA biogenesis and in the degradation of aberrant transcripts. These aberrant transcripts are recognised and targeted for rapid degradation by mRNA surveillance mechanisms. These surveillance mechanisms block the expression of defective mRNAs, which can have deleterious consequences for the cell^14–16^. There are three translation-associated mRNA surveillance pathways that target mRNAs for degradation to prevent the production of potentially toxic proteins. Nonsense-mediated decay (NMD) functions to detect premature stop codons and prevents the expression of truncated proteins. No-go decay (NGD) recognises transcripts on which ribosomes have stalled during translation^17^. Nonstop decay (NSD) is a quality control system for nonstop mRNAs which recognises stalled ribosome at the 3′ end of mRNAs and targets them for rapid degradation^18^. All of these specialised pathways use the same decay enzymes responsible for degrading normal transcripts; however, they differ in the mechanism by which they recognise and target defective mRNAs for degradation^19^.

The importance that mRNA surveillance pathways might have in regulating proteostasis is currently not well understood. The aim of this study was to determine whether loss of NSD, NMD or NGD, would have any effects on proteostasis by examining protein aggregation in strains defective in mRNA surveillance pathways. We show that defects in mRNA quality control systems result in the production of aberrant proteins that tend to misfold and form aggregates. The aggregated proteins share common biophysical properties with aggregation-prone proteins, suggesting that mRNA surveillance pathways normally function to suppress defective protein production arising from errors in translation.

## Results

### Disruption of mRNA surveillance pathways causes widespread protein aggregation

**Figure 1:**
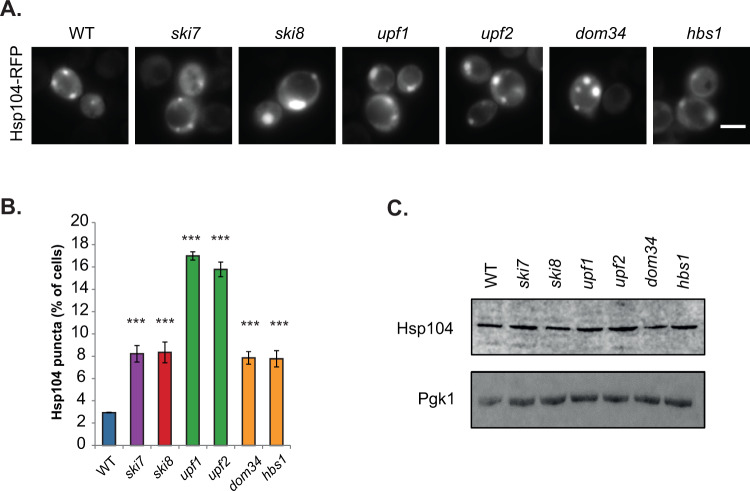
Strains lacking components of mRNA surveillance pathways have higher levels of protein aggregation. (**A**) Hsp104-RFP was visualized in wild-type and mutant strains disrupted for NGD (*dom34, hbs1*), NMD (*upf1, upf2*), NSD (*ski7*) and the Ski complex (*ski8*). Examples of cells containing visible puncta are shown. (**B**) The percentage of cells containing visible Hsp104-RFP puncta is quantified for each strain. Data shown are the means of three independent biological repeat experiments ±SD. Significance is shown compared with the wild-type strain; ***p < 0.001. (**C**) Western blot analysis of Hsp104 protein levels. Blots were probed with a Pgk1 antibody as a loading control. The full blots are shown in Supplementary Fig. 1.

To examine whether protein aggregation occurs following loss of mRNA surveillance pathways, we utilised mutant strains disrupted for NGD (*dom34, hbs1*), NMD (*upf1, upf2*), NSD (*ski7*) and the Ski complex (*ski8*)^20^. We monitored sites of protein aggregation using a fluorescently-tagged Hsp104 chaperone. Hsp104-RFP is predominantly observed as diffuse cytoplasmic fluorescence in cells under normal growth conditions, whereas, it accumulates at the sites of protein aggregation following protein misfolding^21–23^. Diffuse cytoplasmic Hsp104-RFP fluorescence was detected in the majority of wild-type cells examined and only 3% of cells contained visible puncta marking sites of protein aggregation (Fig. 1A and B). In contrast, more puncta were observed in all of the mRNA surveillance mutant strains. The number of Hsp104-RFP puncta detected was elevated approximately three-fold in *ski7, ski8*, *dom34*, and *hbs1* mutant strains, whilst a higher increase was observed in NMD (*upf1, upf2*) mutants with approximately 16–18% of cells examined contained visible Hsp104 puncta (Fig. 1B). Western blot analysis was used to confirm that these differences did not arise due to any differences in Hsp104 expression levels which were similar in the wild-type and mRNA surveillance mutant strains (Fig. 1C and Supplementary Fig. 1).

**Figure 2:**
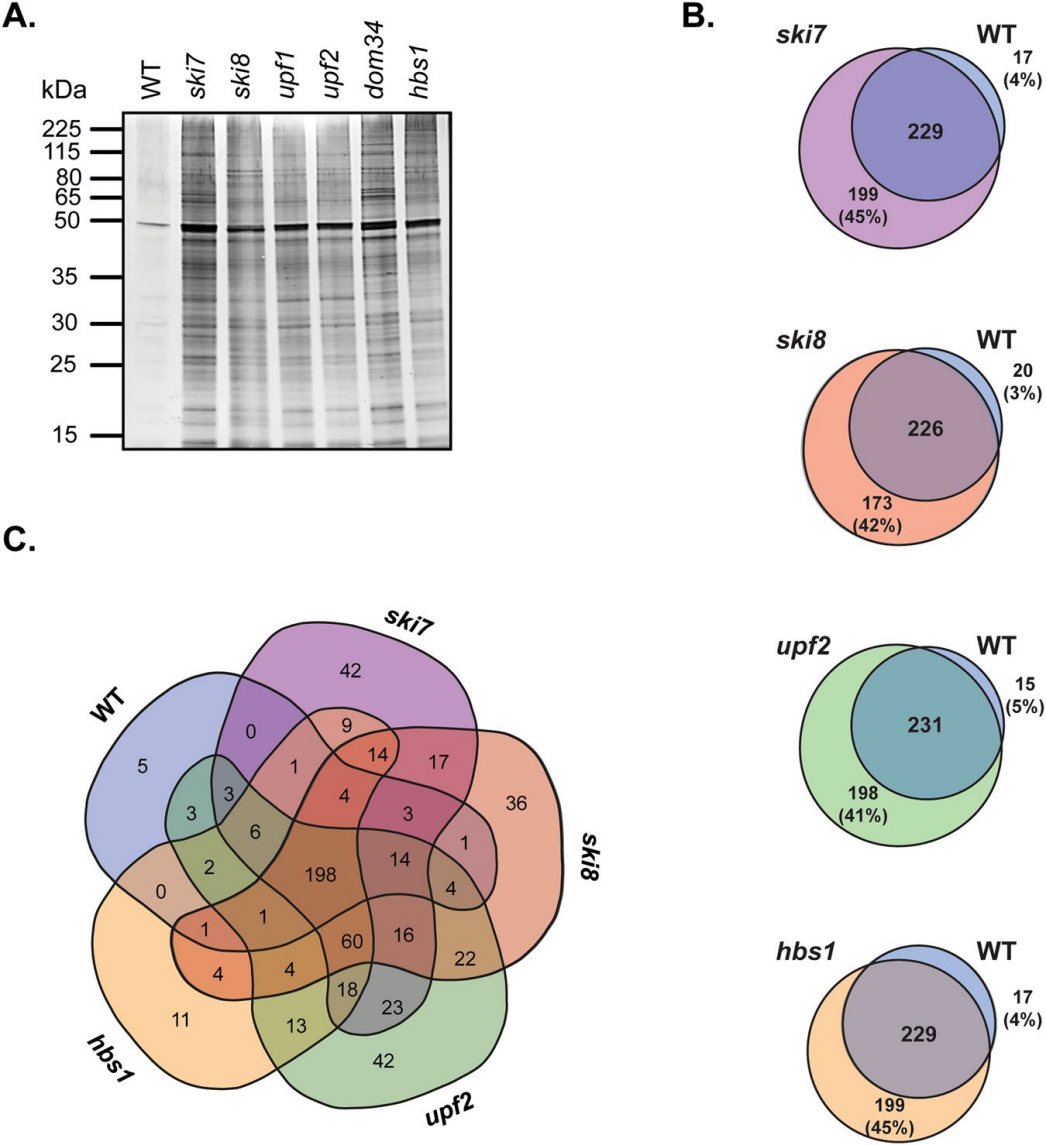
Identification of aggregated proteins in mRNA surveillance mutant strains. (**A**) Protein aggregates were isolated from the wild-type and mRNA surveillance mutant strains and analysed by SDS-PAGE and silver staining. (**B**) Proteins within insoluble aggregate fractions were identified by LC-MS and Venn diagrams show pairwise comparisons of the aggregated proteins in the wild-type and mutant strains. (**C**) Venn diagram comparing the aggregated proteins in the wild-type and mRNA surveillance mutant strains. 198 proteins were identified that aggregate in all five strains and are referred to as the Common-set.

Protein aggregates were purified and visualised using an established biochemical approach that separates insoluble proteins from soluble proteins by differential centrifugation, and removes any contaminating membrane proteins using detergent washes^10,24,25^. Aggregates were prepared from the wild-type and mRNA surveillance mutants, separated using SDS-PAGE and visualized by silver-staining (Fig. 2A). Low levels of protein aggregation were observed in the wild-type strain and increased aggregation was observed in all mRNA surveillance pathway mutants.

### Identification of aggregated proteins in mRNA surveillance mutants

The proteins that aggregate following loss of mRNA surveillance pathways were identified using mass spectrometry. We focused on single NMD (*upf2*) and NGD (*hbs1*) mutants along with *ski7* and *ski8* mutants. Our analysis identified 246 (wild-type), 428 (*ski7*), 399 (*ski8*), 429 (*upf2*) and 428 (*hbs1*) aggregated proteins. More aggregated proteins were identified in the mRNA surveillance mutants compared with the wild-type strain but there was generally a large overlap in the proteins that aggregated in both the wild-type and mutant strains (Fig. 2B). In pair-wise comparisons more proteins were found to be uniquely aggregated in mRNA surveillance mutants (*ski7* = 45%; *ski8* = 42%; *upf2* = 41%; *hbs1* = 45%) compared with the wild-type strain (wild-type = 3–5%). There was also a large overlap (198 proteins) in the proteins that aggregated in all five strains, indicating that most proteins aggregate because they are aggregation-prone rather than arising due to any mutant specific-effects (Fig. 2C). Some unique proteins were identified (*ski7* = 42; *ski8* = 36; *upf2* = 42; *hbs1* = 11) suggesting that a few proteins do aggregate in an mRNA surveillance pathway-specific manner.

### Functional analysis of aggregated proteins in mRNA surveillance mutants

**Figure 3:**
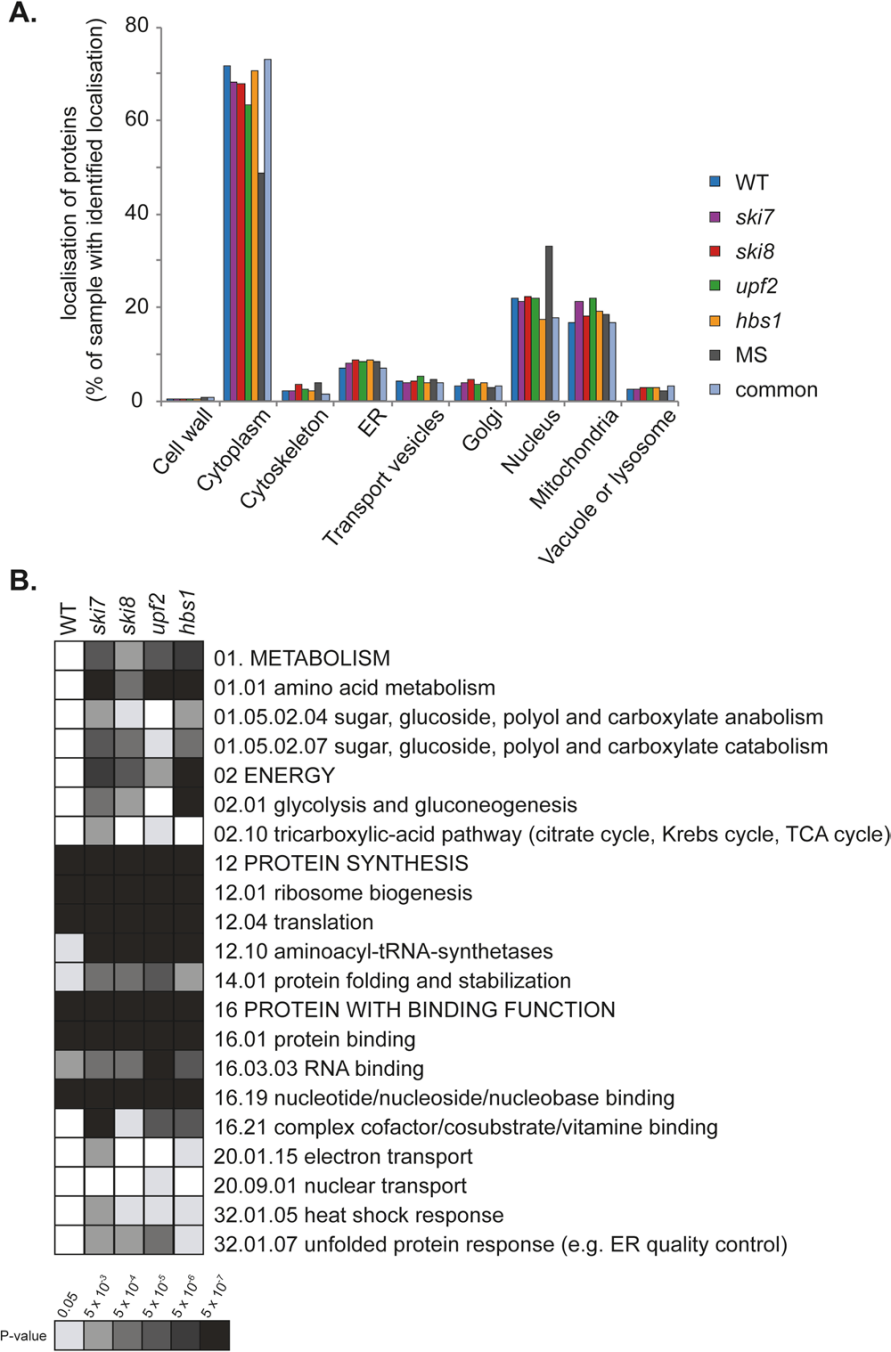
Localisation and functional analysis of aggregated proteins in mRNA surveillance mutants. (**A**) Histogram showing the relative localisation of the proteins present in the aggregates isolated from the wild-type (246), *ski7* (428), *ski8* (399), *upf2* (429) and *hbs1* (349) mutant strains. The localisation of proteins in the MS-set and the common set are also presented for comparison. Proteins with annotated localisation for each cellular component were compared to the total proteins with annotated localisation in each data set. (**B**) MIPS functional categorisation of aggregated proteins identified in the wild-type and mRNA surveillance mutant strains. Significantly enriched functional categories within the data-sets were determined using FunCat. Results are ordered on MIPS category classification numbers and overarching categories are in capitals. Confidence of each classification category is shown as Bonferroni corrected *p*-values.

Given that protein function is generally related to its subcellular localisation, we next examined the localisation of the aggregated proteins in the wild-type and mRNA surveillance mutant strains. For comparison, we used a list of yeast proteins detected by mass spectrometry in logarithmically growing cells, referred to as the MS-set, to represent the properties of unaggregated proteins^26^. No major differences were observed in the proportion of aggregated proteins localised to different compartments in the wild-type and mutant strains. The major fraction of proteins examined were predicted to localise in the cytoplasm of all strains examined (Fig. 3A). Proteins were also predicted to localise to the nucleus, mitochondrion, ER, Golgi, vacuole, cytoskeleton and transport vesicles.

We next analysed the datasets for enrichment of any functional categories to determine whether particular biological processes may be affected by protein aggregation following loss of mRNA surveillance pathways. Significant functional enrichment within the datasets was determined using the MIPS Functional Catalogue^27^. Proteins present in the aggregates isolated from the wild-type (246), *ski7* (428), *ski8* (399), *upf2* (429) and *hbs1* (349) mutant strains were compared. Our analysis revealed that the proteins within the aggregates isolated from all strains could generally be grouped into two major overarching categories: protein synthesis and proteins with binding function or cofactor requirement (Fig. 3B). For mRNA surveillance mutants, proteins in two additional major categories were enriched: metabolism and energy. Additionally, stress categories including the heat shock response and the unfolded protein response were enriched in the mRNA surveillance mutants but not in the wild-type.

### Analysis of the physicochemical properties of aggregated proteins identified in mRNA surveillance mutants

We next assessed the physicochemical properties of the aggregated proteins within our datasets to determine whether they possess particular properties which make them aggregation-prone. For this analysis, we compared the Common-set consisting of 198 proteins which aggregate in all strains including the wild-type (Fig. 2C, middle panel), with the proteins which were found to specifically aggregate in mRNA surveillance mutant strains but not in the wild-type strain (Fig. 2B: *ski7*, n = 199; *ski8*, n = 173; *upf2*, n = 198; *hbs1*, n = 133).

**Figure 4:**
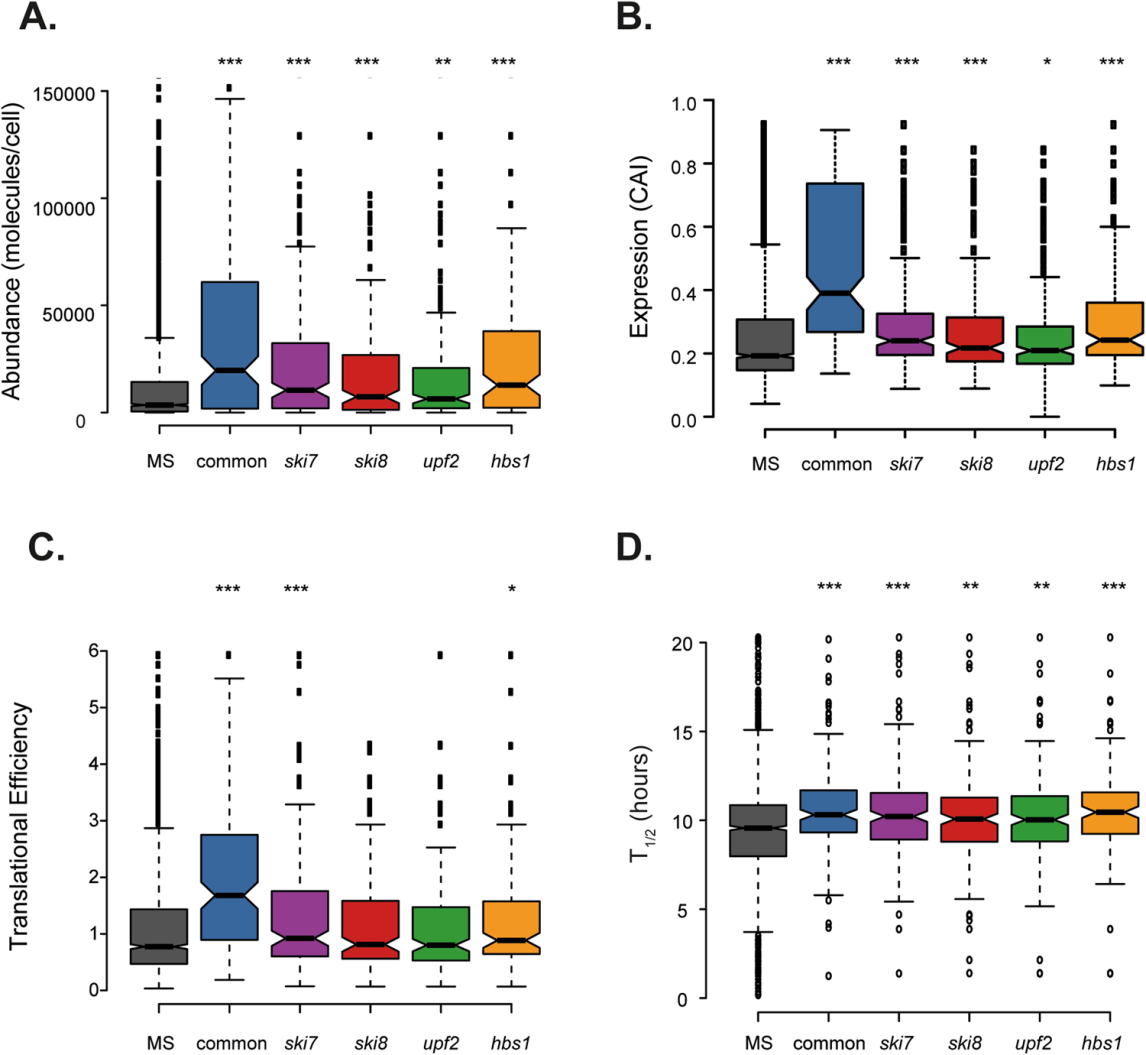
Proteins within aggregate fractions are abundant and highly expressed. Comparison of the aggregated proteins present in the Common-set (198 proteins) with proteins present in mRNA surveillance mutant only sets (*ski7*, n = 199; *ski8*, n = 173; *upf2*, n = 198; *hbs1*, n = 133). Aggregated proteins were compared with a list of unaggregated proteins identified by mass-spectrometry referred to as the MS-set (Washburn *et al*. 2001). (**A**) The abundance of proteins (molecules/cell) in each set during non-stress conditions^47^. (**B**) The codon adaptation index (CAI) as an indicator of gene expression level. (**C**) Tranlsational Efficiency (TE) expressed as the ratio of ribosome footprint density to mRNA density^28^. (**D**) Comparison with protein stability measured as protein half-lives in hours^29^. Mann–Whiney U-tests were used to assess the statistical significance of observed differences: *p < 0.05, **p < 0.01, ***p < 0.001.

Analysis of the aggregated proteins in the Common-set revealed that they are significantly more abundant (as indicated by the number of molecules/cell), more highly expressed (as indicated by a higher codon adaptation index; CAI) and are translated at a higher rate (as determined using translational efficiency measurements^28^) compared with the MS-set (Fig. 4A–C). We used a global proteome turnover database^29^ to compare the stability of the proteins in their native folded states and found that the proteins in the Common-set also show an average longer half-life compared with the proteins in the MS-set (Fig. 4D). Similarly, more highly expressed, abundant and stable proteins were also identified in the aggregate fractions isolated from mRNA surveillance mutant strains, compared with the MS-set (Fig. 4A–D). However, the proteins which aggregated in mRNA surveillance mutants were generally less abundant, less highly expressed and less stable that the proteins in the Common-set suggesting that the threshold for aggregation may be reduced in mRNA surveillance mutants.

**Figure 5:**
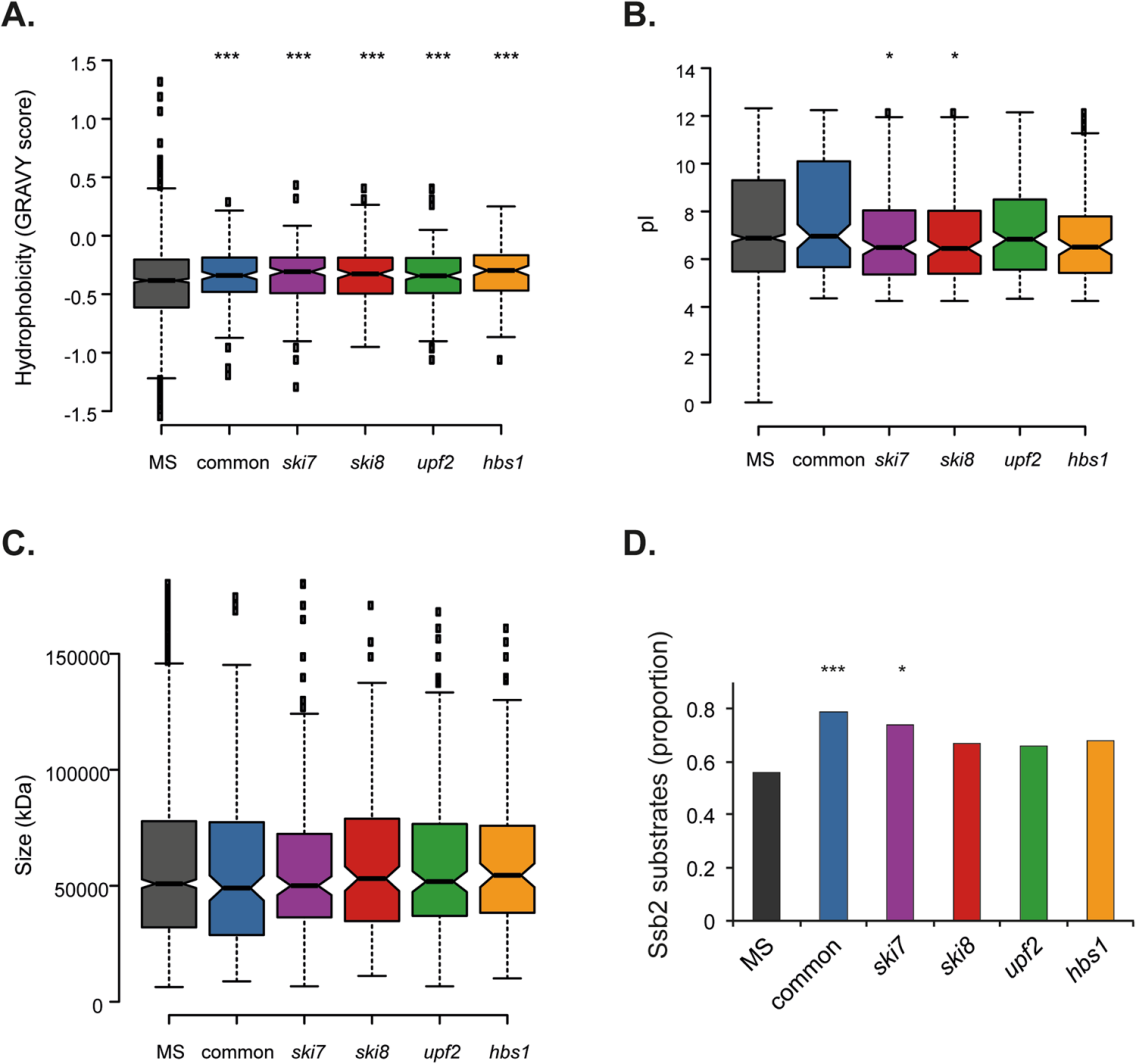
Analysis of physicochemical properties of aggregated proteins. (**A**) Comparison of the grand average of hydrophobicity (GRAVY) scores as a measure of hydrophobicity. (**B**) Comparison of Isoelectric points (pI). (**C**) Comparison of protein size (kDa). Mann–Whiney U-tests were used to assess the statistical significance of observed differences: *p < 0.05, ***p < 0.005. (**D**) Comparison with proteins that are co-translational substrates of Ssb2^32^.

To further examine the aggregated proteins identified in mRNA surveillance mutants, we compared a number of physicochemical properties. Proteins within the Common-set and mRNA surveillance mutant sets were significantly enriched for hydrophobic proteins in agreement with hydrophobicity being a driver of protein aggregation (Fig. 5A). There was no enrichment for proteins with altered pI’s in the Common, *upf2* and *hbs1* sets, whereas, the aggregated proteins identified in the *ski7* and *ski8* sets tended to have lower pI’s. No differences in the sizes (molecular weight) of the aggregated proteins were observed (Fig. 5C).

For comparison, we also examined the physicochemical properties of the uniquely aggregated proteins identified in each of the mRNA surveillance mutants (Fig. 2C: *ski7* = 42; *ski8* = 36; *upf2* = 42;* hbs1* = 11) to determine whether they possess any particular properties which might cause their aggregation to be mutant-specific. No significant enrichments for any functional categories or subcellular localizations were identified in these datasets. It should be noted that this may not be surprising given the relatively small sizes of these datasets. However, unlike the aggregated protein datasets used above (proteins which aggregate in mRNA surveillance mutant strains but not in the wild-type strain using pair-wise comparisons) there was no enrichment for highly expressed (translational efficiency, CAI), abundant (molecules per cell) or stable (T_1/2_) proteins compared with the MS-set (Supplementary Fig. 2A–D). There was also no enrichment for hydrophobic proteins apart from in the *hbs1* mutant, although it should be noted that this dataset only contains 11 proteins (Supplementary Fig. 2E). These data further emphasise the idea that the threshold for proteins to aggregate is reduced in mRNA surveillance compared with a wild-type strain.

### The proteins which aggregate in mRNA surveillance pathways are similar to the proteins that aggregate during nascent protein misfolding and ageing

**Figure 6:**
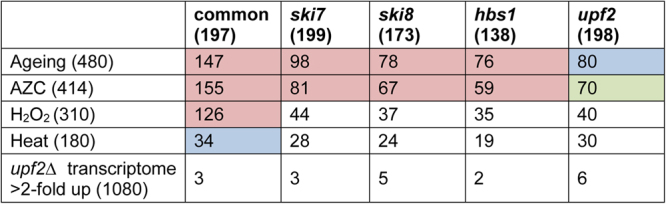
The proteins which aggregate in mRNA surveillance pathways are similar to the proteins which aggregate during nascent protein misfolding and ageing. Comparison of the aggregated proteins present in the Common-set and mRNA surveillance mutant sets with other datasets including proteins that aggregate during postmitotic ageing in yeast^30^, proteins that aggregate during AZC stress^10^, proteins that aggregate during hydrogen peroxide stress^10^, proteins that aggregate during heat-shock^33^ and mRNAs that are increased greater than two-fold in a *upf2* mutant compared with a wild-type strain^34^. Numbers in brackets indicate the size of each dataset. The significance of overlaps was determined by a hypergeometric test. Blue: *p* < 0.05, green: *p* < 0.01, red: *p* < 0.001.

We compared the proteins that aggregate in mRNA surveillance mutants with proteins that aggregate during ageing. Yeast cells have been used as a model for the chronological lifespan of post-mitotic cells and 480 proteins have been identified which aggregate during yeast ageing^30^. We found that there was a significant overlap between the proteins which aggregate during ageing and the Common-set, and also in the proteins that aggregate in the mRNA surveillance pathways suggesting that errors in translation may account for some of the aggregation that occurs during ageing (Fig. 6).

We also compared the proteins that aggregate in mRNA surveillance mutants with proteins that have been shown to aggregate in response to different stress conditions. Nascent protein misfolding can be caused by the proline analogue azetidine-2-carboxylic acid (AZC) which is competitively incorporated into nascent proteins in place of proline. AZC alters the conformation of the polypeptide backbone resulting in decreased thermal stability and misfolding^31^. Significant overlaps were identified between AZC-induced aggregates^10^ and the aggregated proteins in the Common-set and mRNA surveillance pathway mutants suggesting that aggregation may occur during nascent protein misfolding in mRNA surveillance mutants (Fig. 6). We additionally examined whether aggregated proteins are enriched for co-translational substrates of Ssb chaperones using an available Ssb2 data-set^32^. Ssb2, a member of the HSP70 family, is a ribosome-associated molecular chaperone that is involved in the folding of newly-synthesized polypeptide chains. In agreement with the idea that aggregated proteins are susceptible to aggregation during nascent protein folding, the Common-set was significantly enriched in proteins that are co-translational Ssb2 substrates compared to the MS proteome (Fig. 5D). In contrast to AZC stress, no significant overlaps were found between proteins that aggregate in response to oxidative stress conditions caused by hydrogen peroxide exposure^10^ and the proteins that aggregate in mRNA surveillance mutants (Fig. 6). There was however, a significant overlap between the Common-set and the hydrogen peroxide-set (Fig. 6). Similary, no significant overlaps were found between proteins which aggregate in response to acute heat stress conditions^33^ and proteins which specifically aggregate in mRNA surveillance mutants (Fig. 6).

Finally we examined whether there is any correlation between mRNA levels and protein aggregation. We reasoned that if aggregation arises due to translation of mRNAs that are normally degraded in surveillance mutants, we might see an accumulation of these mRNAs in an mRNA surveillance mutant. For this analysis we compared the aggregated proteins with transcripts that have been identified as up-regulated in a *upf2* mutant^34^. However, no correlation was found between the up-regulated mRNAs and the proteins which aggregate in mRNA surveillance mutants including the *upf2* mutant (Fig. 6).

## Discussion

mRNA quality control mechanisms recognise and degrade aberrant mRNAs including mRNAs containing premature stop codons (PTCs), mRNAs lacking termination codons and mRNAs containing structures which promote pausing during translation elongation^19^. We found that increased protein aggregation is a common property of mRNA surveillance mutants suggesting that a failure to degrade these mRNAs results in the production of aberrant proteins. This is similar to recent findings which have shown that disrupting the ribosome-associated quality control (RQC) complex, which recognises stalled proteins and promotes their ubiquitination and degradation, similarly results in protein aggregation^35,36^.

Our analysis was performed using cells grown under non-stress conditions indicating that mRNA surveillance pathways normally act to prevent aberrant mRNAs driving translation-dependent aggregation during normal growth and metabolism. There are a number of possible mechanisms by which loss of surveillance pathways might promote aggregation. For example, aberrant or truncated proteins may be produced by translation of mRNAs which would normally be degraded in surveillance mutants. We did not see any accumulation of mRNAs encoding aggregated proteins in a *upf2* mutant. However, this may simply indicate that any mRNAs that accumulate, may do so at relatively low levels. Alternatively, an accumulation of stalled/aberrant polypeptides in mRNA surveillance mutants could sequester chaperones leading to the aggregation of other proteins. These proteins may aggregate themselves as well as act to nucleate the aggregation of other proteins.

Our analysis identified a significant overlap in the proteins that aggregate in different mRNA surveillance pathway mutants. Not surprisingly therefore, the intracellular localisation of the proteins that aggregate in mRNA surveillance mutants is similar to the localisation of the proteins that aggregate in a wild-type strain. Similarly, the protein functional categories that might be affected as a result of protein aggregation titrating proteins away from their normal soluble forms is predominantly the same in wild-type and mRNA surveillance mutant strains. This is consistent with the idea that the increased protein aggregation detected in mRNA surveillance pathway mutants predominantly arises due to the increased aggregation of a core set of what are already aggregation-prone proteins, rather than aggregation occurring in a mutant-specific manner.

Many studies have identified hydrophobicity as a driving force for protein aggregation. The burial of hydrophobic segments within protein structures prevents exposed hydrophobic regions from interacting with other exposed hydrophobic regions leading to aberrant protein-protein interactions^37–39^. In agreement with this idea, the aggregated proteins identified in mRNA surveillance mutants were commonly enriched for hydrophobicity. Aggregation-prone proteins also tend to be more abundant and highly expressed, compared with unaggregated proteins^10–12^. Similarly, the aggregated proteins identified in mRNA surveillance mutants were enriched for highly translated and abundant proteins. The threat of aggregation may be unavoidable in highly concentrated proteins as they pose a bigger challenge for the proteostasis network, particularly when the proteostasis machinery is compromised. Estimates have suggested that the aggregation tendency of globular proteins may be as high as 20% in diverse proteomes^40^. This may result from the crowded and complex environment that highly expressed nascent proteins encounter resulting in a greater chance of specific and non-specific molecular interactions, making aggregation more likely to occur^41^.

There was a significant overlap in the proteins that aggregate in mRNA surveillance mutants compared with the proteins that aggregate in response to nascent protein misfolding, suggesting that protein aggregation in mRNA surveillance mutants may primarily occur during protein synthesis and folding. The commonly aggregated proteins were also enriched for co-translational Ssb2 substrates further emphasising that protein misfolding may occur co-translationally. Taken together, our data are consistent with the idea that mRNA surveillance pathways normally act to suppress the production of abnormal proteins resulting from aberrant translation events.

The fact that there was a significant overlap with the proteins that aggregate in ageing yeast cells suggests that translation-dependent aggregation may be a particular problem as cells age^42^. Two types of mRNA transcripts may be targets for surveillance pathways: those where genetic features make them a constitutive surveillance target (e.g. because of long 3′ UTRs or PTCs) and those where random damage produces aberrant features that are recognised by surveillance pathways. The balance between these two types of target may shift during ageing where higher levels of random damage may be expected to influence the fidelity of translation^42,43^. There was a strong correlation with the proteins that aggregate in response to nascent protein misfolding, whereas, there was little correlation with the proteins that aggregate following a denaturing stress caused by elevated temperature, further emphasising that aberrant translational events most likely underlie the high levels of aggregation observed in mRNA surveillance mutants. An increased understanding of the roles of mRNA surveillance pathways in protecting against the formation of non-functional RNAs and the subsequent production of abnormal proteins will be important given the established links between the degradation of aberrant mRNAs and human diseases^44–46^.

## Methods

### Strains and growth conditions

Yeast strains used were isogenic derivatives of the wild-type 74D-694 (*MATa ade1–14 ura3–52 leu2–3,112 trp1–289 his3–200*) including *ski7::HIS3*, *ski8::HIS3*, *upf1::HIS3, upf2::HIS3*, *dom34::HIS3 and hbs1::HIS3*. Strains were grown at 30 °C (180 rpm) in SCD medium [2% w/v glucose, 0.17% w/v yeast nitrogen base (Appleton Wood) supplemented with Synthetic Complete (SC) Kaiser amino acid mixes (Formedium, England)].

### Analysis of insoluble protein aggregates

All strains were harvested at the same cell optical density (exponential phase) and Insoluble protein aggregates isolated exactly as previously described^25^. Briefly, 20 ODs of cells were harvested by centrifugation at 4000 rpm, 4 °C for 10 min. Cells were washed in 1 ml of Aggregate lysis buffer (ALB – 50 mM potassium phosphate, 1 mM EDTA, 5% (v/v) glycerol, 1 mM PMSF and 1 × complete mini protease cocktail (Roche). Cells were resuspended in 300 μl ALB and spheroplasts were generated following treatment with lyticase (1 mg/ml) for 30 min at 30 °C. Cell breakage was achieved by sonication (Sonifier 150, Branson; 8 × 5 s, Level 4) and samples were adjusted to equal protein concentrations before isolation of protein aggregates by centrifugation at 13000 rpm, 4 °C for 20 min. Insoluble fractions were resuspended in a buffer containing ALB buffer with 2% (v/v) Igepal CA-630 (Sigma) through sonication (4 × 5 s, Level 4). Samples were centrifuged at 13000 rpm for 20 min at 4 °C and the detergent wash was repeated. Residual detergent was removed by two washes with ALB and the pellet was resuspended by sonication. The final insoluble fraction was resuspended in 80 μl ALB and 20 μl reducing 4 × protein loading buffer, separated by reducing SDS-PAGE (10% gels) and visualised by silver staining using the Bio-Rad silver stain plus kit.

Aggregated proteins were identified by mass spectrometry (performed by the Biomolecular Analysis Core Facility, Faculty of Biology, Medicine and Health, The University of Manchester) in triplicate for each condition. For protein identification, protein samples were run a short distance into SDS-PAGE gels and stained using Instant Blue protein stain (Expedeon). Total proteins were excised, trypsin digested, and identified using liquid chromatography-mass spectrometry (LC-MS). Proteins were identified using the Mascot mass fingerprinting programme (www.matrixscience.com) to search the NCBInr and Swissprot databases. These results were put into Scaffold software, (https://www.proteomesoftware.com/products/free-viewer/) that conducts a statistical validation of the data, at both the peptide and protein levels. We selected proteins that had at least 95% statistical confidence of being present in our samples. Final datasets for each condition were determined by selecting proteins that were identified in at least two of the three replicates.

### Protein and western blot analysis

Protein extracts were electrophoresed under reducing conditions on SDS-PAGE minigels and electroblotted onto PVDF membrane (Amersham Pharmacia Biotech). Bound antibody was visualized using LI-COR Odyssey-FC. Primary antibodies used were Pgk1 (459250, ThermoFisher Scientific) and Hsp104 (ab2924, Abcam).

### Fluorescence microscopy

The plasmid containing fluorescently tagged Hsp104 has been described previously^21^. Cells expressing Hsp104-RFP were washed and immobilised on 10% poly-L-lysine-coated slides. All images were acquired on a Delta Vision (Applied Precision) restoration microscope using a 100 × /NA 1.42 Plan Apo objective and TRITC filter (excitation BP555/28 nm, emission BP617/63) from the Sedat filter set (Chroma). Raw images were then deconvolved using the Softworx software and maximum intensity projections of these deconvolved images are shown in the results. At least 100 cells were visualized in triplicate experiments for each strain and the percentage of cells containing visible puncta scored.

### Statistical analyses

Datasets for each condition were assessed for functional enrichment (*p*-value > 0.01; Bonferroni corrected) of functional categories (MIPS database) using FunCat (available at http://www.helmholtz-muenchen.de/en/ibis). Protein abundance data was retrieved from the PaxDB integrated dataset (available at http://pax-db.org). The proteins that aggregate in aged-yeast, AZC, hydrogen peroxide and heat stressed yeast have been described previously^10,30,33^ and statistical significance of the overlap was evaluated with a hypergeometric test. Venn diagrams and visualization of the distribution of protein hits between conditions were made using Bio Venn (http://www.biovenn.nl). Physicochemical data, translation rates and chaperone interactions were evaluated with pair wise Mann-Whitney-Wilcoxon U-tests using GraphPad Prism.

## Electronic supplementary material

Supplementary Fig. 1 and Fig. 2

Supplementary Table 1
