# Author Correction: Loss of mRNA surveillance pathways results in widespread protein aggregation

**DOI:** 10.1038/s41598-021-95596-1

**Published:** 2021-08-12

**Authors:** Nur Hidayah Jamar, Paraskevi Kritsiligkou, Chris M. Grant

**Affiliations:** 1 Division of Molecular and Cellular Function, School of Biological Sciences, Faculty of Biology Medicine and Health, Manchester Academic Health Science Centre, The University of Manchester, Manchester, M13 9PT UK; 2 School of Biosciences and Biotechnology, Faculty of Science and Technology, Universiti Kebangsaan Malaysia, 43600 Bangi, Malaysia

Correction to: *Scientific Reports* 10.1038/s41598-018-22183-2 published online 01 March 2018

The original version of this Article contained errors. Panels upf1 and dom34 in Figure 1 looked to have originated from the same sample. The Authors now reviewed the original data and for clarity all representative images in Figure 1 have been replaced. Additionally, the Authors recalculated the results shown in Figure 1B using the original data and the graph has also been updated. The original Figure 1 is shown below, for reference:

**Figure 1:**
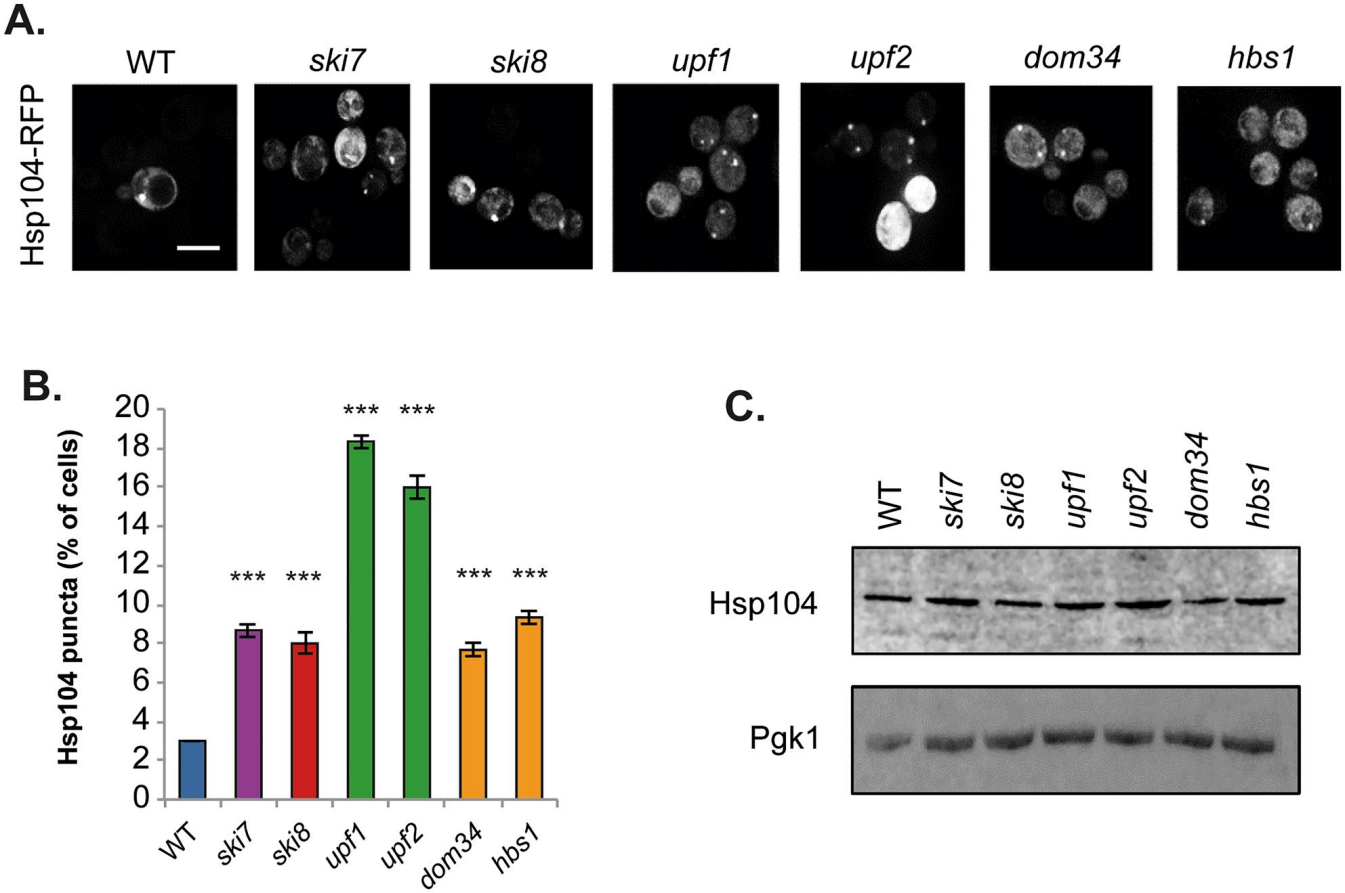
Strains lacking components of mRNA surveillance pathways have higher levels of protein aggregation. (**A**) Hsp104-RFP was visualized in wild-type and mutant strains disrupted for NGD (*dom34, hbs1*), NMD (*upf1, upf2*), NSD (*ski7*) and the Ski complex (*ski8*). Examples of cells containing visible puncta are shown. (**B**) The percentage of cells containing visible Hsp104-RFP puncta is quantified for each strain. Data shown are the means of three independent biological repeat experiments ± SD. Significance is shown compared with the wild-type strain; ***p < 0.001. (**C**) Western blot analysis of Hsp104 protein levels. Blots were probed with a Pgk1 antibody as a loading control. The full blots are shown in Supplementary Fig. 1.

The original version of the Article has been corrected.

