# Supplementary material for: Loss of mRNA surveillance pathways results in widespread protein aggregation
Source: Sci Rep. 2018 Mar 1;8:3894. doi: 10.1038/s41598-018-22183-2 (PMC5832753; doi:10.1038/s41598-018-22183-2)
Supplement: Supplementary file 1 — Supplementary Fig. 1 and Fig. 2 [file 41598_2018_22183_MOESM1_ESM.pdf]

Loss of mRNA surveillance pathways results in widespread protein aggregation

Nur Hidayah Jamar*[1,2], Paraskevi Kritsiligkou*[1] and Chris M. Grant[1,#]

[1] Division of Molecular and Cellular Function, School of Biological Sciences, Faculty of Biology Medicine and Health, Manchester Academic Health Science Centre, The University of Manchester, Manchester, M13 9PT, UK.

[2] School of Biosciences and Biotechnology, Faculty of Science and Technology, Universiti Kebangsaan Malaysia, 43600, Bangi, Malaysia.

*Equal contributions

[#]Corresponding Author: Professor Chris M. Grant,
The University of Manchester,
Faculty of Biology, Medicine and Health,
Division of Molecular and Cellular Function,
The Michael Smith Building,
Oxford Road, Manchester M13 9PT, U.K.
Phone: (0161) 306 4192
Email: chris.grant@manchester.ac.uk

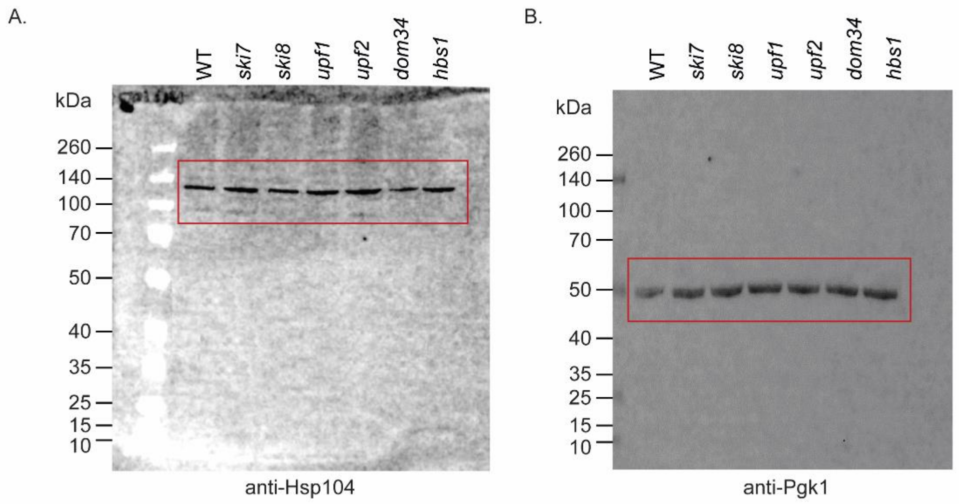

Supplementary Fig. 1. Western blot analysis of Hsp104 and PGK1 in mRNA surveillance mutants. The red boxes indicate the segments of the blots that were cropped and presented in Fig. 1C.

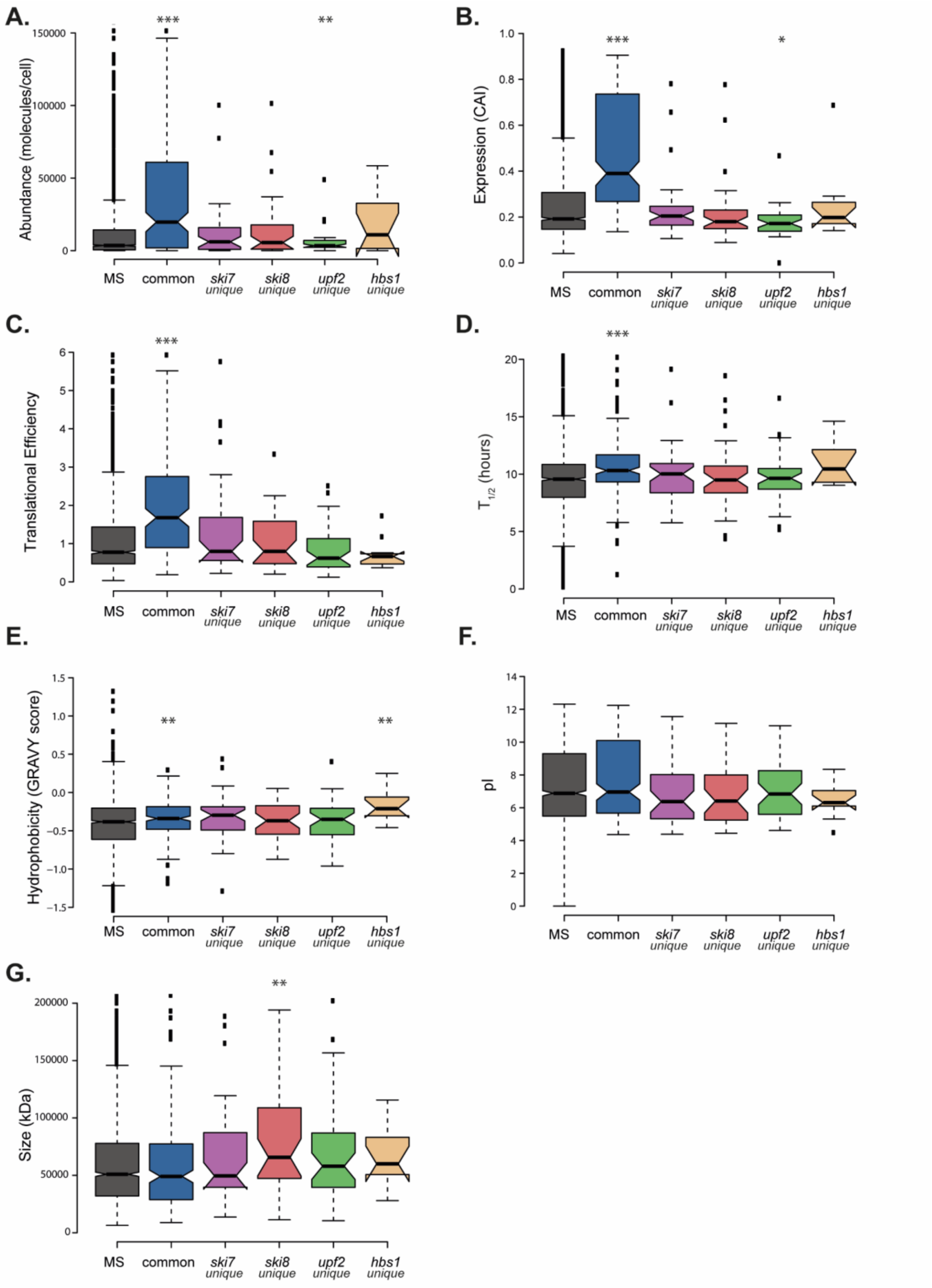

Supplementary Fig. 2. Analysis of physicochemical properties of aggregated proteins. Comparison of the proteins present in the Common-set (198 proteins) with proteins present in mRNA surveillance mutant unique sets (ski7, n=42; ski8, n=36; upf2, n=42; hbs1, n=11). Aggregated proteins were compared with a list of unaggregated proteins identified by mass-spectrometry referred to as the MS-set. **A.** The abundance of proteins (molecules/cell), **B.** The codon adaptation index (CAI), **C.** Translational efficiency, **D.** Protein stability (protein half-lives in hours), **E.** Grand average of hydrophobicity (GRAVY), **F.** Isoelectric points (pI) and **G.** protein sizes (kDa). Mann–Whiney U-tests were used to assess any statistical significances compared with the MS-set: *p < 0.05, **p < 0.01, *** p < 0.001.
